# Direct comparison of the acute subjective, emotional, autonomic, and endocrine effects of MDMA, methylphenidate, and modafinil in healthy subjects

**DOI:** 10.1007/s00213-017-4650-5

**Published:** 2017-05-27

**Authors:** Patrick C. Dolder, Felix Müller, Yasmin Schmid, Stefan J. Borgwardt, Matthias E. Liechti

**Affiliations:** 1 Division of Clinical Pharmacology and Toxicology, Department of Biomedicine, Department of Clinical Research, University Hospital Basel and University of Basel, Schanzenstrasse 55, CH-4056 Basel, Switzerland; 2 Department of Psychiatry (UPK), University of Basel, Basel, Switzerland

**Keywords:** MDMA, Methylphenidate, Modafinil, Emotion recognition, Sexual arousal

## Abstract

**Rationale:**

3,4-Methylenedioxymethamphetamine (MDMA) is used recreationally and investigated as an adjunct to psychotherapy. Methylphenidate and modafinil are psychostimulants that are used to treat attention-deficit/hyperactivity disorder and narcolepsy, respectively, but they are also misused as cognitive enhancers. Little is known about differences in the acute effects of equally cardiostimulant doses of these stimulant-type substances compared directly within the same subjects.

**Methods:**

We investigated the acute autonomic, subjective, endocrine, and emotional effects of single doses of MDMA (125 mg), methylphenidate (60 mg), modafinil (600 mg), and placebo in a double-blind, cross-over study in 24 healthy participants. Acute drug effects were tested using psychometric scales, the Facial Emotion Recognition Task (FERT), and the Sexual Arousal and Desire Inventory (SADI).

**Results:**

All active drugs produced comparable hemodynamic and adverse effects. MDMA produced greater increases in pupil dilation, subjective good drug effects, drug liking, happiness, trust, well-being, and alterations in consciousness than methylphenidate or modafinil. Only MDMA reduced subjective anxiety and impaired fear recognition and led to misclassifications of emotions as happy on the FERT. On the SADI, only MDMA produced sexual arousal-like effects. Only MDMA produced marked increases in cortisol, prolactin, and oxytocin. In contrast to MDMA, methylphenidate increased subjective anxiety, and methylphenidate and modafinil increased misclassifications of emotions as angry on the FERT. Modafinil had no significant subjective drug effects but significant sympathomimetic and adverse effects.

**Conclusions:**

MDMA induced subjective, emotional, sexual, and endocrine effects that were clearly distinct from those of methylphenidate and modafinil at the doses used.

**Electronic supplementary material:**

The online version of this article (doi:10.1007/s00213-017-4650-5) contains supplementary material, which is available to authorized users.

## Introduction

3,4-Methylenedioxymethamphetamine (MDMA, ecstasy) is used recreationally and investigated as an adjunct to psychotherapy for patients with posttraumatic stress disorder (Mithoefer et al. 2010; Oehen et al. 2013). Methylphenidate and modafinil are stimulants that are used to treat attention-deficit/hyperactivity disorder and narcolepsy, respectively, but also misused as cognitive enhancers (Liakoni et al. 2015; Maier et al. 2015; Maier et al. 2013; Repantis et al. 2010). The neurotransmitter interaction profile of MDMA clearly differs from classic psychostimulants (Liechti 2015). MDMA releases serotonin and oxytocin (Francis et al. 2016; Hysek et al. 2012c; Kirkpatrick et al. 2014b; Simmler et al. 2013), in contrast to psychostimulant amphetamines (Liechti 2015; Simmler et al. 2013) and related substances, such as methylphenidate and modafinil, that enhance dopaminergic and noradrenergic neurotransmission without affecting serotonin or oxytocin (Hannestad et al. 2010; Madras et al. 2006; Qu et al. 2008; Schmeichel and Berridge 2013; Schmid et al. 2014; Simmler et al. 2014; Zolkowska et al. 2009). The main goal of the present study was to characterize and compare the effects of MDMA with the stimulant-type substances methylphenidate and modafinil at single relatively high doses matched for their cardiostimulant properties (comparable rate-pressure products; Hysek et al. 2014b; Jasinski 2000).

The acute effects of MDMA have been extensively studied in healthy subjects (Dumont and Verkes 2006; Vizeli and Liechti 2017) but only rarely compared directly with other orally administered stimulant substances (Hysek et al. 2014b; Parrott et al. 2011; Schmid et al. 2014; Tancer and Johanson 2003). MDMA induced greater “empathogenic” mood effects, including closeness to others, openness, trust, happiness, and wanting to be with others, compared with oral methylphenidate (Hysek et al. 2014a; Schmid et al. 2014). MDMA, but not methylphenidate, impaired the recognition of sad and fearful emotions on the Face Emotion Recognition Task (FERT) and increased biomarkers of serotonergic activity, including plasma concentrations of cortisol, prolactin, and oxytocin (Bedi et al. 2010; Hysek et al. 2014a; Kirkpatrick et al. 2014b; Kuypers et al. 2017; Schmid et al. 2014). In contrast, methylphenidate, but not MDMA, increased subjective concentration (Hysek et al. 2014a; Schmid et al. 2014) and sexual arousal that was elicited by explicit visual sexual stimuli (Schmid et al. 2015b). MDMA produced greater drug liking, elation, and good drug effects than amphetamine (Tancer and Johanson 2003), whereas other studies reported positive mood following methamphetamine but not MDMA administration (Parrott et al. 2011). However, these studies used only a few outcome measures and did not comprehensively evaluate emotional drug effects. MDMA may have unique empathogenic and prosocial effects that are distinct from classic stimulants (Bedi et al. 2010; Bershad et al. 2016; Hysek et al. 2014b; Kamilar-Britt and Bedi 2015; Schmid et al. 2014), but these differences need to be confirmed using blinded administration of MDMA and different stimulants within the same subjects. Therefore, the present cross-over study directly compared the subjective, emotional, autonomic, sexual, and endocrine effects of MDMA, methylphenidate, and modafinil. We expected MDMA to produce acute effects that are distinct from those of methylphenidate and modafinil. The a priori hypotheses defined in the study protocol were that MDMA, but not methylphenidate or modafinil, at the doses used, will produce prosocial and empathic feelings (closeness to others, openness, trust, want to be with other people) and elevate plasma levels of oxytocin (Hysek et al. 2014a; Hysek et al. 2014b; Schmid et al. 2014).

The acute effects of modafinil and methylphenidate are also different. Amphetamine and methylphenidate have greater subjective effects than modafinil (Franke et al. 2017; Jasinski 2000; Makris et al. 2007). In contrast, modafinil may have greater wakefulness-promoting effects than methylphenidate (Jasinski 2000; Repantis et al. 2010). In addition to comparisons with MDMA, the present study directly compared the effects of the neuroenhancers methylphenidate and modafinil in healthy subjects, complementing a previous study in stimulant users (Jasinski 2000).

## Materials and methods

### Study design

We used a double-blind, placebo-controlled, randomized, cross-over design with four experimental sessions (125 mg MDMA, 60 mg methylphenidate, 600 mg modafinil, and placebo) in 24 subjects. The order of the four experimental sessions was counterbalanced. The washout periods between sessions were at least 7 days. The study was conducted in accordance with the Declaration of Helsinki and approved by the Ethics Committee of Basel and the Swiss Agency for Therapeutic Products (Swissmedic). All of the subjects provided written consent before participating in the study, and they were paid for their participation. The study was registered at ClinicalTrials.gov (NCT01951508).

### Participants

Twenty-four healthy subjects (12 men, 12 women), mean ± SD age of 22.6 ± 3.0 years (range, 19–29 years), were recruited from the University of Basel. The inclusion criteria were 18–45 years old and body mass index of 18–27 kg/m^2^. Subjects with a personal or first-degree-relative history of psychiatric disorders or chronic or acute physical illness were excluded. Additional exclusion criteria were tobacco smoking (>10 cigarettes/day) and a lifetime history of using illicit drugs more than five times, with the exception of occasional cannabis use in the past. In contrast to similar studies in other laboratories, the majority of the participants had never used MDMA or other illicit drugs with the exception of cannabis (Kirkpatrick et al. 2014a; Kuypers et al. 2017). Four participants had used MDMA (one–three times), two had used cocaine on a single occasion, one had used LSD once, one psilocybin once, and three methylphenidate (once or twice). Four had never used cannabis, 11 had used cannabis 1–20 times, and 9 participants had used cannabis >20 times ranging from once/month to 4 times per week. Subjects who used any illicit drugs, including cannabis, within the past 2 months or during the study period were excluded. We performed drug tests at screening and before each test session using TRIAGE 8 (Biosite, San Diego, CA, USA). All female subjects used oral contraceptives and were investigated during the follicular phase of their menstrual cycle (day 2–14 after the start of the menstruation) to account for cyclic changes in reactivity, which has been demonstrated for amphetamines (White et al. 2002).

### Study procedures

The study included a prescreening telephone interview, a screening visit, four experimental sessions (test days), and an end-of-study visit. The experimental sessions began at 8:45 AM. An indwelling intravenous catheter was placed in an antecubital vein for blood sampling, and baseline measurements were performed. MDMA, methylphenidate, modafinil, or placebo was administered at 9:45 AM. Autonomic and subjective effects were assessed repeatedly throughout the session. Blood was collected to determine endocrine effects and substance concentrations. A functional magnetic resonance imaging (fMRI) scan was performed at 11:15 AM–12:15 PM during the expected drug peak effects (Hysek et al. 2014b; Schmid et al. 2014; Wong et al. 1998). The fMRI findings will be published elsewhere. Face emotion recognition was assessed at 12:15 PM. A standardized small lunch was served at 1:15 PM, and the subjects were sent home at 3:45 PM.

### Study drugs

±MDMA hydrochloride (Lipomed AG, Arlesheim, Switzerland) was prepared as gelatin capsules with mannitol as the filler. Identical placebo (only mannitol) capsules were prepared. MDMA was administered in a single absolute dose of 125 mg corresponding to a relatively high dose of (mean ± SD) 1.9 ± 0.3 mg/kg body weight. This dose of MDMA is in the high range of the doses typically used in clinical research (Kirkpatrick et al. 2015; Kirkpatrick et al. 2014a; Kuypers et al. 2017) but also used in clinical studies in patients with PTSD (Mithoefer et al. 2010; Oehen et al. 2013) and is within the dose range that is used recreationally (Brunt et al. 2012). Immediate-release methylphenidate tablets (10 mg, Ritalin, Novartis AG, Rotkreuz, Switzerland) were encapsulated within opaque gelatin capsules, and identical placebo capsules were prepared. The therapeutic starting dose of methylphenidate is 10 mg and average therapeutic doses are 20–30 mg/day. Methylphenidate was administered in a single relatively high dose of 60 mg (Korostenskaja et al. 2008; Martin et al. 1971). The subjective and cardiostimulant effects of this dose have previously been assessed on the same tests (Hysek et al. 2014b) and have also been statistically compared with a lower dose of 40 mg (Schmid et al. 2014). The doses of MDMA and methylphenidate used were expected to be equivalent regarding their cardiovascular stimulant effects (Hysek et al. 2014b). Modafinil tablets (100 mg, Teva Pharma AG, Basel, Switzerland) were encapsulated within opaque gelatin capsules, and identical placebo capsules were prepared. The therapeutic starting dose of modafinil is 100 mg and common doses are 400 mg/day. Modafinil was administered in a single high dose of 600 mg. The goal was to use high single doses of all substances with comparable cardiostimulant effects (Hysek et al. 2014b; Jasinski 2000) and to maximize the subjective drug effects.

### Measures

#### Autonomic effects

Blood pressure, heart rate, and tympanic body temperature were repeatedly measured 1 h before and 0, 0.3, 0.6, 1, 1.25, 1.5, 2.5, 3, 4, 5, and 6 h after drug administration as previously described in detail (Hysek et al. 2010). The peak rate-pressure product (systolic blood pressure × heart rate) was the main autonomic outcome measure reflecting the maximal total hemodynamic response to a substance and expected to be comparable across substances at the doses used (Hysek et al. 2014b; Jasinski 2000).

#### Subjective effects

Subjective effects were assessed repeatedly using visual analog scales (VASs) 1 h before and 0, 0.3, 0.6, 1, 1.25, 1.5, 2.5, 3, 4, 5, and 6 h after drug administration. The VASs included “any drug effect,” “good drug effect,” “bad drug effect,” “drug liking,” “happy,” “concentration,” “open,” “trust,” “feeling close to others,” “I want to be with other people,” and “I want to hug someone” (Hysek et al. 2014a). The VASs were presented as 100-mm horizontal lines (0–100%), marked from “not at all” on the left to “extremely” on the right. The 60-item Adjective Mood Rating Scale (AMRS; (Janke and Debus 1978)) and State-Trait Anxiety Inventory (STAI; (Spielberger et al. 1970)) were administered 1 h before and 1.25, 2.5, and 5 h after drug administration. The German version of the 49-item Addiction Research Center Inventory (ARCI; (Bopp et al. 2005; Martin et al. 1971)) was administered 1 h before and 1.25 h after drug administration. The 5-Dimensions of Altered States of Consciousness (5D–ASC) scale (Dittrich 1998; Studerus et al. 2010) was administered 5 h after drug administration to retrospectively rate the effects of the drugs.

#### Endocrine effects

The plasma levels of prolactin, cortisol, oxytocin, and vasopressin were measured at baseline and 1.5 and 2.5 h after drug administration and analyzed as described previously (Hysek et al. 2012b; Neumann et al. 2013).

### Facial emotion recognition task

We used the FERT, which is sensitive to the effects of MDMA (Bedi et al. 2010; Hysek et al. 2014b; Kirkpatrick et al. 2014b; Schmid et al. 2014) and methylphenidate (Hysek et al. 2014b; Schmid et al. 2014). The task included 10 neutral faces and 160 faces that expressed one of four basic emotions (i.e., happiness, sadness, anger, and fear), with pictures morphed between 0% (neutral) and 100% in 10% steps. Two female and two male pictures were used for each of the four emotions. The stimuli were presented in random order for 500 ms and then were replaced by the rating screen where participants had to indicate the correct emotion. The main outcome measure was accuracy (proportion correct). Additionally, we analyzed whether incorrectly identified emotional expressions were misclassified as neutral or other emotions (Bedi et al. 2010; Schmid et al. 2014). The FERT was performed 2.5 h after drug administration.

### Sexual arousal and desire Inventory

The SADI is a self-report scale that includes 54 items (adjectives or short sentences) and measures subjective sexual arousal and desire (Persson et al. 2016; Toledano and Pfaus 2006). Each item is rated from 0 (“does not describe it at all”) to 5 (“describes it perfectly”). The questionnaire includes four overlapping factors: “Evaluative,” “Physiological,” “Negative/Aversive,” and “Motivational.” The “Evaluative” scale consists of 27 items that describe cognitive and emotional aspects (i.e., tempted, passionate, seductive, attractive). The “Physiological” scale consists of 17 items that describe autonomic reactions to sexual arousal (i.e., sensitive to touch, stimulated, excited, heart beats faster). The “Negative/Aversive” scale consists of 17 items that describe aspects that are opposite to sexual arousal (i.e., anxious, displeasure, repulsion, angry). The “Motivational” scale consists of 10 items that are related to the motivation to engage in sexual activity (i.e., driven, urge to satisfy, horny, impatient). The SADI was administered 1 h before and 3 h after drug administration.

### Substance plasma concentrations

The plasma levels of methylphenidate, modafinil, MDMA, and the MDMA metabolites 3,4-methylenedioxyamphetamine (MDA) and 4-hydroxy-3-methoxymethamphetamine (HMMA) were determined 1 h before and 1, 1.5, 2.5, 3, 4, and 6 h after substance administration using liquid chromatography-mass spectrometry/mass spectrometry as previously described (Hysek et al. 2012a; Hysek et al. 2014b). The maximal plasma concentration (*C*_max_) and time to *C*_max_ (*T*_max_) were obtained directly from the observed concentration-time curves.

### Adverse effects

Adverse effects were assessed 1 h before and 6 h (acute) and 24 h (sub-acute) after drug administration using the 66-item List of Complaints (Zerssen 1976). The scale yields a total adverse effects score and reliably measures physical and general discomfort.

### Statistical analyses

Repeated measures are expressed as peak effects or peak changes from baseline (*E*_max_). The data were analyzed using repeated-measures analysis of variance (ANOVA), with drug as the within-subjects factor, followed by Tukey post hoc comparisons based on significant main effects. Some of the VASs data were not normally distributed and were therefore analyzed using Friedman ANOVAs with drug as the within-subject factor, followed by Wilcoxon matched pairs tests. The criterion for significance was *p* < 0.05. The criterion was adjusted for the multiple comparisons within the VAS and the AMRS using the Bonferroni method.

## Results

All 24 participants completed all sessions of the study. Autonomic, subjective, and endocrine peak effects and statistics are shown in Table 1 and Supplementary Table S1.

**Table 1:** Comparison of the acute effects of MDMA, methylphenidate, modafinil, and placebo (*N* = 24)

		Placebo	MDMA	Methylphenidate	Modafinil		
		(mean ± SEM)	(mean ± SEM)	(mean ± SEM)	(mean ± SEM)	*F* 3.69	*p*=
Autonomic effects							
Systolic blood pressure (mm Hg)	*E*_max_	135 ± 3.0	152 ± 3.2***	145 ± 3.1***#	143 ± 3.4**##	20.41	<0.001
Diastolic blood pressure (mm Hg)	*E*_max_	78.8 ± 1.6	88.1 ± 1.8***	84.0 ± 1.8**#	85.7 ± 2.0***	13.24	<0.001
Heart rate (beats/min)	*E*_max_	73.1 ± 2.5	90.3 ± 3.0***	91.6 ± 3.6***	87.9 ± 3.5***	23.82	<0.001
Rate pressure product (beats·mmHg/min)	*E*_max_	9603 ± 383	13,377 ± 536***	12,985 ± 625***	12,273 ± 667***	26.15	<0.001
Body temperature (°C)	*E*_max_	37.3 ± 0.1	37.6 ± 0.1**	37.6 ± 0.1**	37.9 ± 0.1***	12.55	<0.001
Pupil size (mm)	*E*_max_	6.5 ± 0.1	7.4 ± 0.1***	6.7 ± 0.1**###	6.7 ± 0.2*###	76.11	<0.001
Pupil size after light stimulus (mm)	*E*_max_	4.7 ± 0.1	6.4 ± 0.2***	4.8 ± 0.1###	4.8 ± 0.1###	148.78	<0.001
Constriction amplitude (mm)	*E*_min_	1.8 ± 0.1	0.86 ± 0.10***	1.8 ± 0.1###	1.7 ± 0.1###	34.47	<0.001
Subjective effects							
Visual Analog Scale (VAS, %max)							
Any drug effect	ΔE_max_	10.0 ± 2.4	75.3 ± 5.1***	32.8 ± 6.8*###	23.7 ± 5.2###	45.51	<0.001
Good drug effect	ΔE_max_	7.5 ± 2.5	73.2 ± 5.3***	35.5 ± 7.7**###	23.9 ± 5.4###	45.68	<0.001
Bad drug effect	ΔE_max_	4.2 ± 3.3	12.7 ± 3.5	11.5 ± 4.5	10.3 ± 4.9	10.49	NS
Drug liking	ΔE_max_	9.4 ± 3.0	75.5 ± 5.5***	36.3 ± 8.2*###	24.8 ± 5.4###	40.9	<0.001
Happy	ΔE_max_	2.7 ± 1.2	31.0 ± 3.6***	10.8 ± 3.6##	8.1 ± 2.4###	41.06	<0.001
Concentration	ΔE_max_	−6.5 ± 1.5	−29.3 ± 3.4	−11.6 ± 3.6	−13.3 ± 3.6	9.56	NS
Open	ΔE_max_	2.4 ± 1.0	19.6 ± 3.7**	11.2 ± 3.5*	9.5 ± 2.9	20.08	<0.01
Trust	ΔE_max_	1.5 ± 0.9	24.5 ± 4.1**	8.0 ± 3.0##	8.6 ± 3.7#	24.85	<0.001
Feeling close to others	ΔE_max_	1.1 ± 0.8	18.5 ± 3.8**	9.7 ± 2.6*#	5.7 ± 2.1#	24.79	<0.001
I want to be with other people	ΔE_max_	3.1 ± 1.9	21.0 ± 4.3***	10.1 ± 3.5#	9.9 ± 3.8#	18.46	<0.01
I want to hug someone	ΔE_max_	0.6 ± 0.6	15.1 ± 3.6*	7.0 ± 3.3	3.3 ± 2.3#	23.13	<0.001
Adjective Mood Rating Scale (AMRS score)							
Well-being	ΔE_max_	0.04 ± 0.37	5.8 ± 1.0***	2.5 ± 0.7#	1.3 ± 0.7##	14.52	<0.001
Emotional excitation	ΔE_max_	−0.13 ± 0.56	3.0 ± 0.9*	2.6 ± 0.8	1.2 ± 0.7	4.91	<0.05
Activity	ΔE_max_	0.79 ± 0.45	2.0 ± 0.6	2.5 ± 0.5	0.50 ± 0.4	3.23	NS
Extroversion	ΔE_max_	0.0 ± 0.4	2.3 ± 0.6**	1.3 ± 0.5	1.36 ± 0.5	5.25	<0.05
Introversion	ΔE_max_	0.50 ± 0.29	2.4 ± 0.5*	1.3 ± 0.4	0.9 ± 0.4	4.36	<0.05
Concentration	ΔE_max_	0.3 ± 0.32	0.46 ± 0.46	1.13 ± 0.42	−0.33 ± 0.25	3.05	NS
State-Trait Anxiety Inventory (state anxiety score)							
	*E*_max_	36.6 ± 1.0	37.6 ± 1.4	42.0 ± 1.8*	39.5 ± 1.6	3.25	<0.05
	*E*_min_	32.8 ± 0.9	29.0 ± 1***	34.5 ± 1.0###	32.5 ± 1.2#	6.58	<0.001
Sexual Arousal and Desire Inventory							
Evaluative		1.4 ± 1.3	19.5 ± 4.3***	6.5 ± 3.4#	8.4 ± 3.6#	6.98	<0.001
Negative/Aversive		0.04 ± 1.3	−0.09 ± 1.5	0.3 ± 1.4	4.9 ± 2.6	1.88	NS
Physiological		1.6 ± 0.9	13.2 ± 2.4***	6.6 ± 2.4#	5.0 ± 2.2##	8.03	<0.001
Motivational		0.4 ± 0.6	4.8 ± 1.5*	1.1 ± 1.2	2.5 ± 1.4	3.22	<0.05
Hormones							
Cortisol (nmol/L)	*E*_max_	630 ± 53.5	942 ± 41.5***	648 ± 37.7**###	653 ± 40.5###	35.55	<0.001
Prolactin (mU/L)	*E*_max_	334 ± 32.7	1086 ± 143***	295 ± 30.9###	288 ± 24.0###	30.46	<0.001
Oxytocin (pg/mL)	*E*_max_	5.0 ± 1.5	58.7 ± 7.9***	6.3 ± 1.3###	4.29 ± 0.87###	44.90	<0.001
Vasopressin (pg/mL)	*E*_max_	4.1 ± 0.3	4.4 ± 0.5	4.23 ± 0.67	3.44 ± 0.27	2.15	NS

VAS and AMRS *P* values were Bonferroni adjusted values considering the multiple subscales used (11 and 6 for the VAS and AMRS, respectively)

*NS* not significant, *Emax* maximal effect, *ΔEmax* maximal difference from baseline

*p < 0.05, **p < 0.01, ***p < 0.001 compared with placebo; #*p* < 0.05, ##*p* < 0.01, ###*p* < 0.001 compared with MDMA

### Autonomic effects

**Fig. 1:**
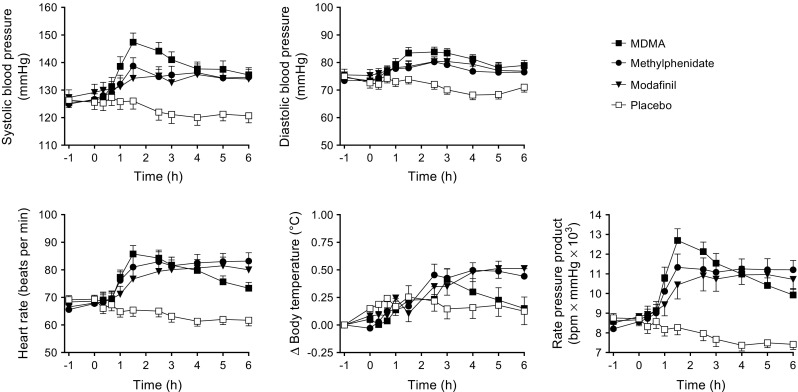
Autonomic responses to MDMA, methylphenidate, modafinil, and placebo. All active treatments increased systolic and diastolic blood pressure, heart rate, and body temperature. MDMA produced slightly higher increases in blood pressure than methylphenidate and modafinil. However, the overall hemodynamic response, expressed as the rate pressure product, similarly increased after all active treatments compared with placebo. The data are expressed as the mean ± SEM in 24 subjects. The substances were administered at *t* = 0

Drug effects on vital signs over time are shown in Fig. 1, and peak effects are shown in Table 1. All active substances produced comparable and significant increases in body temperature and similar hemodynamic stimulation, considering the similar increase in the rate-pressure products compared with placebo. MDMA markedly and significantly increased pupil size in the dark and after a light stimulus and decreased the constriction amplitude. Methylphenidate and modafinil produced only very small but significant increases in pupil size in the dark. MDMA produced significantly greater alterations in pupil function than methylphenidate and modafinil, including markedly greater pupil size in the dark and significantly lower responses to a light stimulus (Supplementary Fig. S1, Table 1).

### Subjective effects

**Fig. 2:**
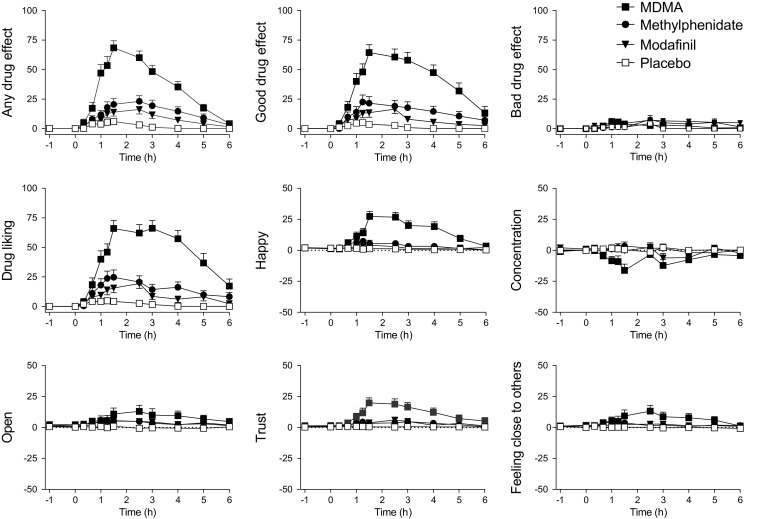
Subjective effects of MDMA, methylphenidate, modafinil, and placebo on the VASs. MDMA produced greater subjective effect ratings on most VASs compared with methylphenidate and modafinil. MDMA produced greater any drug effects, good drug effects, drug liking, happiness, trust, and feeling close to others than methylphenidate and modafinil. None of the substances produced significant bad drug effects compared with placebo. Modafinil did not produce any significant subjective effects compared with placebo. The data are expressed as the mean ± SEM in 24 subjects. The substances were administered at *t* = 0

**Fig. 3:**
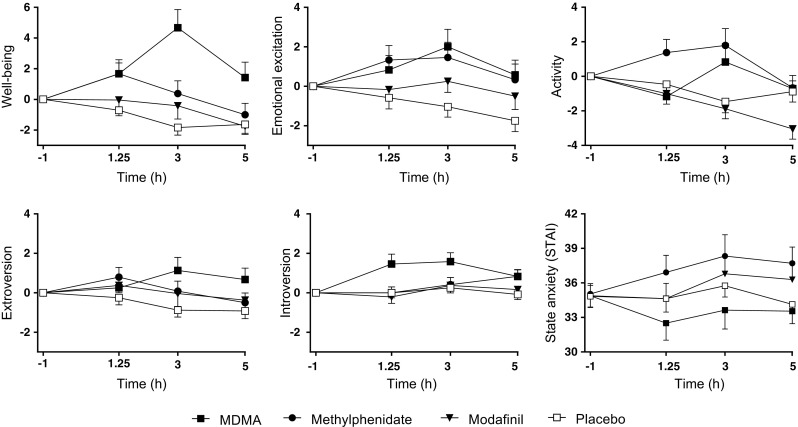
On the AMRS, MDMA significantly increased ratings of well-being, emotional excitation, extroversion, and introversion. Methylphenidate and modafinil produced no effects on any of the AMRS scales. On the STAI, MDMA decreased state anxiety, and methylphenidate increased state anxiety. The data are expressed as the mean ± SEM in 24 subjects. The substances were administered at *t* = 0

Subjective drug effects over time are shown in Fig. 2 and Fig. 3, and peak responses are shown in Table 1 and Supplementary Table S1. On the VASs (Fig. 2), MDMA increased ratings for any drug effect, good drug effect, drug liking, happiness, open, trust, feeling close to others, I want to be with other people, and I want to hug someone compared with placebo. Methylphenidate produced significant increases in any drug effect, good drug effect, and drug liking but only very small increases in ratings of open, and feeling close to others compared with placebo. Modafinil produced no significant subjective effects compared with placebo. MDMA increased ratings for any drug effect, good drug effect, drug liking, happy, trust, feeling close to others, and want to be with other people more than methylphenidate or modafinil. On the AMRS (Fig. 3), MDMA increased well-being, emotional excitation, extraversion, and introversion compared with placebo. Methylphenidate and modafinil had no effects on the AMRS. MDMA increased well-being more than methylphenidate or modafinil. On the STAI (Fig. 3), MDMA reduced state anxiety, methylphenidate increased anxiety, and modafinil had no effect. On the ARCI (Supplementary Table S1), MDMA increased amphetamine-group, morphine, and pentobarbital-chlorpromazine-alcohol group ratings compared with placebo. Methylphenidate increased amphetamine and benzedrine group ratings compared with placebo. Modafinil had no significant effects on the ARCI. MDMA increased morphine and pentobarbital-chlorpromazine-alcohol group ratings significantly more than methylphenidate and modafinil. On the 5D-ASC scale (Supplementary Table S1), MDMA produced effects on oceanic boundlessness and anxious ego dissolution. Methylphenidate and modafinil had no effects on any of the 5D-ASC scales.

### Endocrine effects

**Fig. 4:**
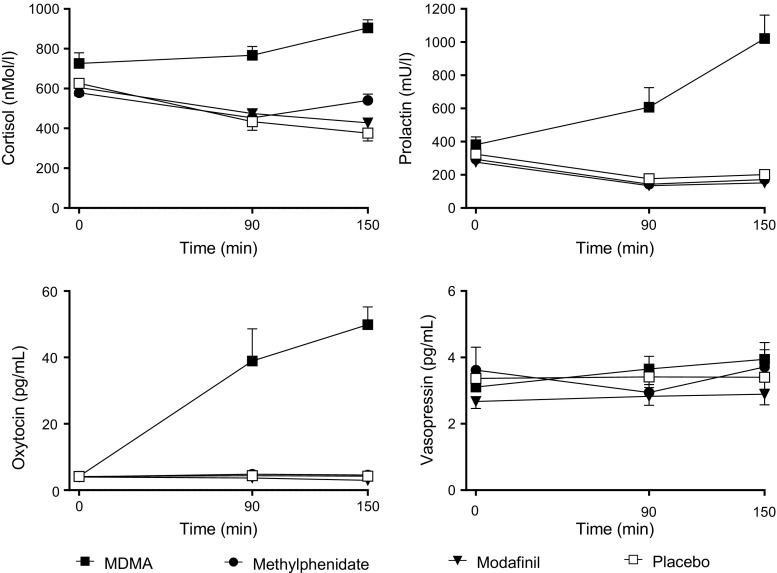
Endocrine effects of MDMA, methylphenidate, modafinil, and placebo. MDMA increased levels of cortisol, prolactin, and oxytocin compared with placebo. Methylphenidate produced a weak but significant increase in cortisol compared with placebo. Modafinil had no endocrine effects. The data are expressed as the mean ± SEM in 24 subjects. The substances were administered at *t* = 0

MDMA increased plasma concentrations of cortisol, prolactin, and oxytocin compared with placebo and all of the other active treatments (Fig. 4, Table 1). Methylphenidate produced a slight increase in cortisol concentrations, and modafinil had no effect. None of the active treatments increased vasopressin concentrations.

### Facial emotion recognition

**Fig. 5:**
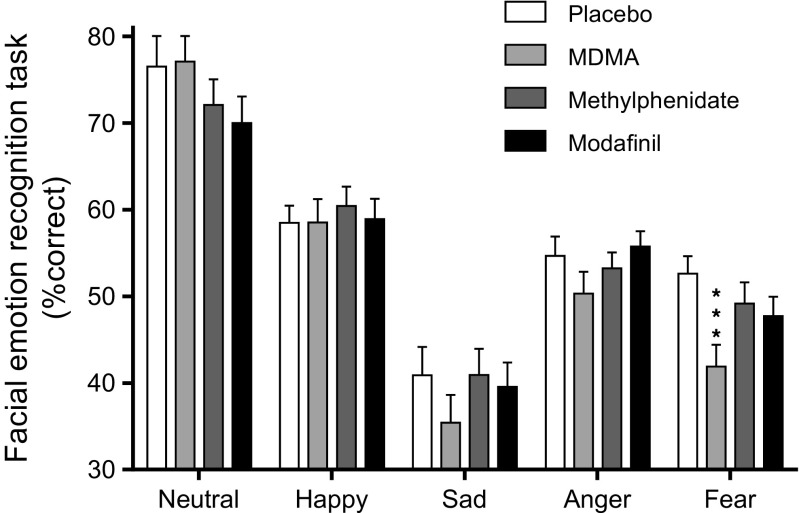
On the Facial Emotion Recognition Task, MDMA significantly impaired the recognition of fearful faces compared with placebo. Methylphenidate and modafinil did not significantly alter emotion recognition. The data are expressed as the mean ± SEM in 22 subjects

The effects of MDMA, methylphenidate, and modafinil on the FERT are shown in Fig. 5. Complete datasets were unavailable for two subjects because of technical reasons. The ANOVA revealed a significant main effect of drug on the decoding accuracy of fearful faces (*F*_3,63_ = 7.38 *p* < 0.001). The post hoc tests showed that MDMA significantly impaired the recognition of fearful faces compared with placebo (*p* < 0.001), methylphenidate (*p* < 0.01), and modafinil (*p* < 0.05). There was a significant main effect of drug on misclassification of emotions as happy (*F*_3,63_ = 3.35 *p* < 0.05). MDMA significantly increased the misclassification of emotions as happy compared with placebo (*p* < 0.05). In contrast, methylphenidate (*p* < 0.05) and modafinil (*p* < 0.05) increased misclassifications of emotions as angry (main effect of drug: *F*_3,63_ = 3.38 *p* < 0.05). However, methylphenidate and modafinil did not alter emotion recognition accuracy for any of the emotions.

### Sexual arousal and desire

Data from one subject were missing. Only MDMA increased scores on the “Evaluative” (*p* < 0.001), “Physiological” (*p* < 0.001), and “Motivational” (*p* < 0.05) scales compared with placebo, but it did not influence scores on the “Negative/Aversive” scale (Table 1). MDMA produced significantly higher ratings on the “Physiological” factor items “tingly all over,” “sensitive to touch,” “enthusiastic,” “warm all over,” “flushed,” “heart beats faster,” and “seductive” compared with placebo. MDMA produced significantly higher ratings on the “Evaluative” factor items “enthusiastic,” “warm all over,” “passionate,” “sensual,” “pleasure,” “heart beats faster,” “happy,” “powerful,” and “forget about all else” compared with placebo MDMA produced significantly higher ratings on the “Motivational” factor item “anticipatory” compared with placebo.

### Substance plasma concentrations

The concentration-time curves for methylphenidate, modafinil, MDMA, MDA, and HMMA are shown in Supplementary Fig. S2. The *C*_max_ values for MDMA, MDA, and HMMA were 192 ± 8.7, 9.1 ± 0.4, and 69.1 ± 10.0 ng/ml, respectively. The *T*_max_ values were 3.7 ± 0.3, 5.7 ± 0.2, and 4.2 ± 0.3 h, respectively. The *C*_max_ values for methylphenidate and modafinil were 27.0 ± 2.1 and 13.2 ± 0.5 μg/ml, respectively. The *T*_max_ values were 3.2 ± 0.3 and 3.3 ± 0.3 h, respectively.

### Adverse effects

On the List of Complaints, MDMA, methylphenidate, and modafinil produced significant and comparable acute adverse effects (up to 6 h) compared with placebo (Supplementary Table S1). The most frequent acute complaints were lack of appetite, dry mouth, and headache. Only modafinil produced significant adverse effects that lasted up to 24 h (mostly insomnia, headache, and lack or appetite). There were no severe adverse effects.

## Discussion

The main finding of the present study was that oral administration of MDMA produced acute subjective, emotional, sensual/sexual, and endocrine effects that were clearly distinct from those of the stimulant drugs methylphenidate and modafinil at oral doses that produced comparable sympathomimetic stimulant effects. The acute effects of MDMA (Dumont et al. 2009; Farre et al. 2004; Hysek et al. 2011; Kirkpatrick et al. 2014b; Liechti et al. 2001; Vollenweider et al. 1998), methylphenidate (Hysek et al. 2014b; Schmid et al. 2015b; Schmid et al. 2014), and modafinil (Jasinski 2000; Rush et al. 2002; Turner et al. 2003; Wong et al. 1999) have been previously described in healthy subjects. The present study further compared their acute responses within the same study and within the same subjects, thus providing direct and valid comparisons. The use of four drug conditions in the within-subject study eliminated any between-subject or between-study differences in comparisons of drug characteristics, and all drugs were administered blind in both active and passive control conditions. The primary research question was whether MDMA is simply a stimulant or whether it has unique namely prosocial and empathogenic effects that are distinct from other stimulants (Bershad et al. 2016). The present study clearly indicated that MDMA has a different effect profile than the stimulant methylphenidate at the doses used, supporting previous, albeit less rigorous, study findings (Bershad et al. 2016; Hysek et al. 2014b; Schmid et al. 2015b; Schmid et al. 2014). Specifically, MDMA increased well-being, good drug effects, drug liking, happiness, trust, feelings of closeness to others, and wanting to be with others, and reduced state anxiety compared with methylphenidate and modafinil at the doses used. The distinct mood effects of MDMA were congruent with its effects on the FERT, including reductions of fear recognition and more misclassifications of emotions as happy. MDMA, but not methylphenidate, has previously been shown to reduce fear recognition on the FERT (Bedi et al. 2010; Hysek et al. 2014b; Kirkpatrick et al. 2014b; Schmid et al. 2014). Additionally, MDMA, but not methylphenidate or modafinil, strongly increased endocrine markers of acutely increased serotonergic activity, including cortisol and prolactin (Seifritz et al. 1996; Sommers et al. 1994; Strajhar et al. 2016). MDMA, but not methylphenidate or modafinil, also markedly increased pupil size at rest and after light stimulation, which is consistent with a previous study (Hysek and Liechti 2012) and other serotonergic substances (Schmid et al. 2015a). On the ARCI, methylphenidate and modafinil produced significantly fewer sedating effects than MDMA, which is consistent with their greater stimulant-type properties.

Pharmacologically, MDMA (Hysek et al. 2012c; White et al. 1996), methylphenidate (Han and Gu 2006; Rothman et al. 2001; Schmeichel and Berridge 2013), and modafinil (Madras et al. 2006; Rowley et al. 2014) all stimulate the noradrenergic system and consistently produced overall similar cardiostimulant effects in the present study. Additionally, MDMA releases serotonin (Hysek et al. 2012c; White et al. 1996) and oxytocin (Dumont et al. 2009; Hysek et al. 2012b; Hysek et al. 2014a; Kuypers et al. 2017; Schmid et al. 2014; Thompson et al. 2007), whereas methylphenidate and modafinil act as dopamine uptake inhibitors (Madras et al. 2006; Rothman et al. 2001). Consistent with the different pharmacological profiles of these substances, MDMA exerted distinct subjective effects across all of our rating scales (VASs, AMRS, ARCI, 5D-ASC, and SADI) compared with methylphenidate and modafinil at the doses used. Consistent with the present study, we previously reported greater well-being, happiness, extroversion, and feelings of closeness after 125 mg MDMA administration compared with 60 mg methylphenidate (Hysek et al. 2014b) and after 75 mg MDMA administration compared with 40 mg methylphenidate (Schmid et al. 2014).

In the present study, MDMA increased circulating concentrations of cortisol, prolactin, and oxytocin as previously shown for different doses of MDMA (Harris et al. 2002; Hysek et al. 2012b; Hysek et al. 2014a; Joyce et al. 1986; Kirkpatrick et al. 2014b; Mas et al. 1999; Schmid et al. 2014; Seibert et al. 2014). MDMA did not alter plasma concentrations of vasopressin, which is consistent with previous studies that reported no change in the concentrations of the vasopressin precursor copeptin (Hysek et al. 2014a; Schmid et al. 2014). In contrast, higher levels of vasopressin following MDMA administration were reported in some earlier studies (Forsling et al. 2001; Henry et al. 1998), and one study reported higher concentrations of the vasopressin precursor copeptin in females (Simmler et al. 2011). Methylphenidate only slightly increased plasma concentrations of cortisol in the present study, which is consistent with no or weak effects of methylphenidate that were reported in other studies (Hysek et al. 2014b; Joyce et al. 1986; Schmid et al. 2014; Seibert et al. 2014). Modafinil had no effects on plasma cortisol, prolactin, oxytocin, or vasopressin concentrations, which is consistent with previous reports for cortisol (Brun et al. 1998). Altogether, the main endocrine finding of the present study was that only MDMA produced marked increases in cortisol, prolactin, and oxytocin concentrations, which is consistent with similar effects of other serotonergic substances (Schmid et al. 2015a; Seibert et al. 2014; Seifritz et al. 1996; Strajhar et al. 2016).

The present study used a relatively high dose of modafinil (600 mg), which did not produce significant or relevant subjective effects despite pronounced cardiostimulant and adverse effects, including insomnia, that lasted up to 24 h. Similarly, modafinil produced no subjective mood effects at doses of 100–400 mg (Franke et al. 2017; Muller et al. 2013; Scoriels et al. 2011; Turner et al. 2003). However, modafinil increased misclassifications of emotions as angry on the FERT. Higher subjective anxiety and aggression were reported after administration of 100 and 200 mg modafinil in young (Randall et al. 2003) but not middle-aged (Randall et al. 2004) healthy volunteers. Modafinil improved the recognition of sad faces in psychotic patients (Scoriels et al. 2011), a finding that was not replicated in the present study in healthy subjects. Consistent with its use as a promoter of wakefulness, 100 and 200 mg modafinil increased alertness in healthy subjects in another study (Turner et al. 2003). Compared with the present study that used a relatively high dose of 600 mg, modafinil had moderate hemodynamic effects at doses of 100–200 mg (Turner et al. 2004; Turner et al. 2003).

In contrast to modafinil, methylphenidate produced significant subjective good drug effects and drug liking and produced moderate increases in feeling open and close to others compared with placebo. In contrast to the effects of MDMA, methylphenidate increased STAI state anxiety and tended to increase subjective concentration, which is consistent with its stimulant properties and use as a cognitive enhancer (Liakoni et al. 2015; Maier et al. 2015; Maier et al. 2013).

In the present study and at the doses used, MDMA, but not methylphenidate or modafinil, increased ratings of sexual arousal and desire on the SADI. This finding was unexpected because dopaminergic stimulants, including cocaine, methamphetamine, and methylphenidate, have been previously shown to increase sexual desire and arousal (Frohmader et al. 2010; Rawson et al. 2002; Schmid et al. 2015b; Semple et al. 2002). Interestingly, intravenous (Volkow et al. 2007) but not oral (Volkow et al. 2007) administration of methylphenidate enhanced self-reported sexual desire, which is consistent with the lack of sexual stimulant effects of oral methylphenidate that was observed in the present study. We previously reported that methylphenidate, but not MDMA, increased sexual arousal that was elicited by explicit visual sexual stimuli (Schmid et al. 2015b). The SADI is very different from the test that was used in the previous study. No stimuli were presented, and subjects simply rated the intensity of various sensations that are typically related to sexual stimulation but without a specific sexual context or related stimuli. MDMA increased many sensations that comprise the “Evaluative” and “Physiological” factors of the SADI, including ratings of enthusiastic, happy, sensual, pleasure, warm all over, and heart beats faster, in a potentially nonspecific sexually related manner. Although the subjects were instructed to make ratings on the SADI specifically with regard to sexual arousal and desire, their ratings may have been confounded by direct somatic and subjective drug effects of MDMA that are unrelated to sexual stimulation. Thus, one may argue that MDMA produced many physiological effects that also coincide with sexual arousal. Other studies reported increases in sexual arousal after methylphenidate but not MDMA administration (Frohmader et al. 2010; Schmid et al. 2015b; Volkow et al. 2007). MDMA users report inconsistent effects on sexual arousal and desire (McElrath 2005; Passie et al. 2005; Theall et al. 2006). MDMA appears to enhance pleasurable sensations, touch, and physical closeness rather than actual sexual engagement, and it reportedly impairs sexual performance (Frohmader et al. 2010; Passie et al. 2005; Theall et al. 2006; Zemishlany et al. 2001). Remaining unclear is whether MDMA produces actual sexual arousal or heightened motivation to engage in sexual activity.

The present study has limitations. We mainly used only one dose of each substance. It is difficult if not impossible to compare active substances if only one dose is used. Thus, the observed differences between drugs were seen at the doses used in this study but may not be present at different doses. However, dose-effect relationships show *E*_max_ curve characteristics (Hysek et al. 2012c) and we used single but relatively high doses of all drugs expected to result in subjective drug effects close to *E*_max_ based on previous studies (Hysek et al. 2012a; Hysek et al. 2012c). MDMA was used at an average dose of 1.9 mg/kg resulting in high mean peak plasma concentrations of 192 ng/ml clearly above the EC_50_ values of 44 and 93 ng/ml MDMA for the cardiostimulant and subjective effects, respectively (Hysek et al. 2012a; Hysek et al. 2012c). Thus, it can be assumed that near-maximal effects were reached at the MDMA dose used in the present study. Similarly, the single doses of methylphenidate and modafinil were high (sixfold the therapeutic starting doses for both substances), and plasma concentrations of methylphenidate and modafinil were twofold higher compared to those in another study using lower doses (Franke et al. 2017). Importantly, all of the drugs produced comparable overall sympathomimetic stimulation as reflected by the similar increase in the peak rate-pressure product and consistent with our attempt to match the doses based on previous data (Hysek et al. 2014b; Jasinski 2000) to produce similar cardiovascular effects. The distinct effects of MDMA and the other two stimulants were seen at the doses used in the present study. However, similarly distinct profiles were reported for MDMA and methylphenidate using identical and also lower doses of both substances and including also dose-response analyses (Hysek et al. 2014b; Schmid et al. 2014). The plasma concentration-time curves of all substances and metabolites measured in the present study were in the expected range based on pharmacokinetic data from other studies (de la Torre et al. 2000; Wong et al. 1998). The *C*_max_ was similar, but the mean *T*_max_ (3.2 h) of methylphenidate was longer compared with our previous study (2.3 h) (Hysek et al. 2014b). Importantly, *T*_max_ values were comparable for all substances used in the present study. Nevertheless, it should be acknowledged that comparisons across substances could be biased by differences in dose and pharmacokinetics.

Another issue relates to the use of many psychometric scales in the present study with the intention to more comprehensively and sensitively describe and compare the effects of the psychoactive substances. This may have increased the risk of change findings. However, the primary study hypothesis of greater socioemotional effects of MDMA compared to methylphenidate and modafinil at the doses used was confirmed. However, the differential effects of the substances on the STAI, ARCI, and SADI should be regarded as exploratory.

In conclusion, MDMA produced acute subjective, emotional, and endocrine effects that were distinct from those of methylphenidate and modafinil at the doses used, which is consistent with their different pharmacological profiles. Modafinil produced acute sympathomimetic stimulation that was comparable to methylphenidate and MDMA, but modafinil produced no emotional or subjective effects at the relatively high dose used.

## Electronic supplementary material

Table S1 Subjective and adverse effects of MDMA, methylphenidate, modafinil, and placebo (*N* = 24). (XLSX 15 kb)

Figure S1 MDMA markedly increased pupil size in the dark and after a light stimulus and reduced pupillary constriction in response to light. Methylphenidate and modafinil only slightly increased pupil size in the dark and had no effect on pupillary constriction in response to light. The data are expressed as the mean ± SEM in 24 subjects. The substances were administered at *t* = 0. (GIF 66 kb)

High Resolution Image (TIFF 157 kb)

Figure S2 Plasma concentration vs. time profiles of methylphenidate, modafinil, MDMA, and the MDMA metabolites 3,4-methylenedioxyamphetamine (MDA) and 4-hydroxy-3-methoxymethamphetamine (HMMA). Peak plasma levels were reached 3.2 ± 0.3 h, 3.3 ± 0.3 h, and 3.7 ± 0.3 h after methylphenidate, modafinil, and MDMA administration, respectively. The data are expressed as the mean ± SEM in 24 subjects. The substances were administered at *t* = 0. (GIF 38 kb)

High Resolution Image (TIFF 106 kb)
