# Supplementary figures and images for: Direct comparison of the acute subjective, emotional, autonomic, and endocrine effects of MDMA, methylphenidate, and modafinil in healthy subjects
Source: Psychopharmacology (Berl). 2017 May 27;235(2):467–79. doi: 10.1007/s00213-017-4650-5 (PMC5813072; doi:10.1007/s00213-017-4650-5)

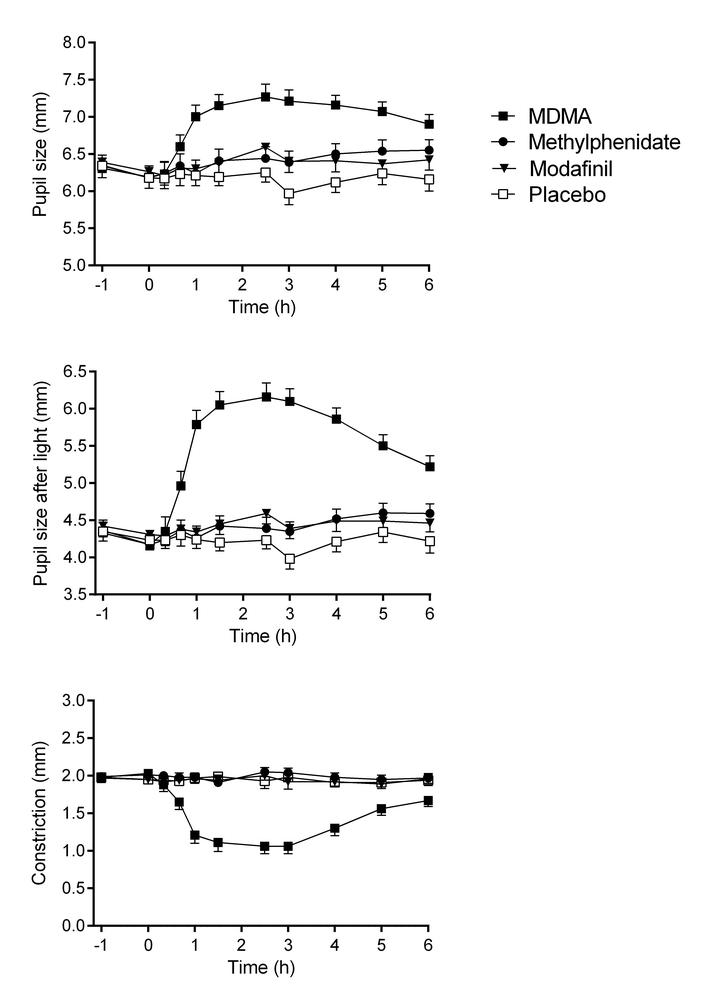

Supplement: Supplementary file 2 — MDMA markedly increased pupil size in the dark and after a light stimulus and reduced pupillary constriction in response to light. Methylphenidate and modafinil only slightly increased pupil size in the dark and had no effect on pupillary constriction in response to light. The data are expressed as the mean ± SEM in 24 subjects. The substances were administered at t = 0. (GIF 66 kb) [file 213_2017_4650_MOESM2_ESM.gif]

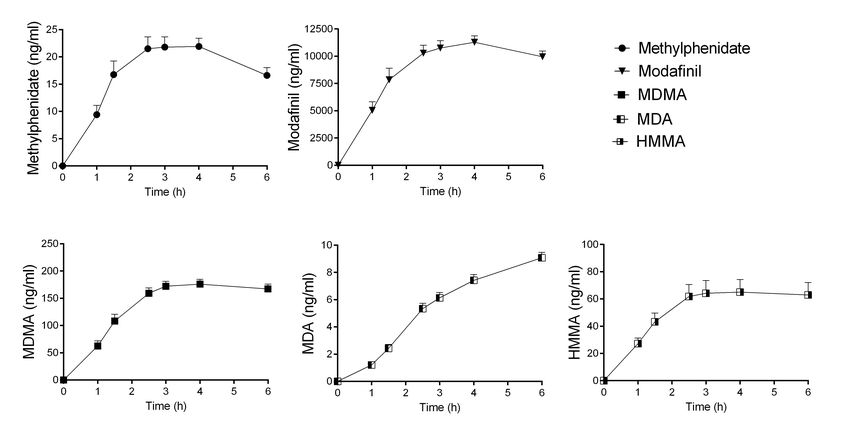

Supplement: Supplementary file 4 — Plasma concentration vs. time profiles of methylphenidate, modafinil, MDMA, and the MDMA metabolites 3,4-methylenedioxyamphetamine (MDA) and 4-hydroxy-3-methoxymethamphetamine (HMMA). Peak plasma levels were reached 3.2 ± 0.3 h, 3.3 ± 0.3 h, and 3.7 ± 0.3 h after methylphenidate, modafinil, and MDMA administration, respectively. The data are expressed as the mean ± SEM in 24 subjects. The substances were administered at t = 0. (GIF 38 kb) [file 213_2017_4650_MOESM4_ESM.gif]
